# Evaluation of Remotely Sensed Inundation Data Sets to Estimate Flood‐Associated Emergency Department Visits After Hurricane Harvey

**DOI:** 10.1029/2025GH001516

**Published:** 2025-12-27

**Authors:** Balaji Ramesh, Julia M. Gohlke, Benjamin Zaitchik, Ayaz Hyder, Jeffrey J. Wing, Gia Barboza‐Salerno, Samarth Swarup

**Affiliations:** ^1^ College of Public Health The Ohio State University Columbus OH USA; ^2^ Population Health Sciences Virginia Maryland College of Veterinary Medicine Virginia Tech Blacksburg VA USA; ^3^ Morton K. Blaustein Department of Earth and Planetary Sciences Johns Hopkins University Baltimore MD USA; ^4^ College of Social Work The Ohio State University Columbus OH USA; ^5^ Biocomplexity Institute and Initiative University of Virginia Charlottesville VA USA

**Keywords:** floods, risk assesment, remote sensing, intestinal infectious diseases, disaster recovery, hurricane Harvey

## Abstract

Floods can increase the risk of adverse health outcomes through multiple pathways, including contamination of food and water. Remotely sensed (RS) inundation extents can help identify regions with expected heightened flood‐related health risks, but variations across inundation data sets and their integration into health risk assessments may affect intervention targeting. We examined if the association between census tract (CT) flooding and intestinal infectious disease related emergency department (IID‐ED) visits differed by RS‐based exposure estimation methods. Two Hurricane Harvey Inundation data sets with different spatiotemporal resolutions were used to estimate CT‐level exposure as percent land flooded and percent population flooded, yielding four exposure variables. These were linked to ED visits by residential CT, and the effect estimates for association between IID‐ED visits and flooding were derived. A 10% increase in land flooded was associated with a 6% (1%–10%) higher risk of IID‐ED visits, while percent population flooded was not significantly associated with IID‐ED visits. No statistically significant differences were found in the effect estimates between the inundation data sets or the exposure representation methods. Combining data sets to identify flooded CTs improved model fitness compared to using either alone, indicating a 1.30 (1.16–1.45) times greater risk of IID‐ED visits in flooded CTs compared to non‐flooded CTs. CTs where the data sets disagreed also showed a 25% (8%–10%) higher risk of IID‐ED visits compared to the mutually agreed non‐flooded CTs. Combining remotely sensed inundation data sets of different specifications can address limitations of individual products and improve identifying intervention areas to mitigate flood‐related health risks.

## Plain Language Summary

Satellite images can identify flooded areas, where residents are more likely to be exposed to contaminants that may cause health problems. However, the specific areas identified vary depending on the configuration of the satellite data used. In this study, we analyzed two satellite‐derived data sets, both capturing flooding from Hurricane Harvey but with different spatial accuracy and observation frequency. We used statistical models to compare emergency room visits for intestinal infectious diseases (IID) between flooded and non‐flooded regions identified by each data set. Each data set independently showed an increased risk of IID‐related emergency department (ED) visits in their identified flooded regions. When the two data sets were combined, they more accurately identified areas at elevated risk. Based on the combined data, individuals in flooded areas had a 30% higher likelihood of visiting the emergency room for IID. We found that a 10% increase in flooded land area was associated with a 6% increase in IID‐related emergency visits. Incorporating population differences within flooded areas did not explain additional variations in IID‐related emergency department visits. These findings suggest that merging multiple satellite data sets can enhance the identification of high‐risk areas, thereby improving the targeting of public health interventions during and after flood events.

## Key Points

- Model fit for flooding‐intestinal infectious disease ED visits association improved when remotely sensed inundation data sets were combined
- The risk of IID ED visits was elevated in areas where inundation data sets disagreed on flooding compared to agreed non‐flooded areas
- Merging high‐spatial/low‐temporal with low‐spatial/high‐temporal inundation data improves identification of areas with elevated IID risk

## 1 Introduction

The frequency of extreme precipitation events has increased since the 1970s, in the United States and globally (Kunkel et al., 2013; Tabari, 2020). This trend is driven by rising global temperatures, which cause more moisture to be held in the atmosphere, resulting in more intense precipitation (Trenberth, 2011; Witze, 2018). At the same time, population dynamics, such as disproportionate population increase in floodplains relative to other places, rapid urbanization, and migration into flood plains driven by economic opportunities have further increased the number of people exposed to floods (Andreadis et al., 2022; Swain et al., 2020; Tellman et al., 2021; Wang et al., 2025). This growing exposure to floods will in turn increase the burden of flood‐related health outcomes such as acute intestinal illness, respiratory illness, skin infections, pregnancy complications, exacerbation of asthma, adverse cardiovascular outcomes, and vector‐borne diseases during and following floods (CDC, 2021b; Coalson et al., 2021; Du et al., 2010; Ghosh et al., 2022; Reacher et al., 2004; Saulnier et al., 2017; Solomon et al., 2006; Xiao et al., 2019).

### 1.1 Flooding and Intestinal Infectious Disease

Increased incidence of intestinal infectious diseases (IID) is commonly reported after floods (Ahern et al., 2005; Alderman et al., 2012; Huang et al., 2023; Saulnier et al., 2017). Some of the pathways through which this occurs include ingestion of contaminated food or water, displacement of people, disruption of sanitation facilities, saturation of subsoil and mobilization of pathogens into ground water, and water treatment infrastructure damage leading to contaminated public drinking water supply (Levy et al., 2016). Some of these pathways, such as ingestion of contaminated water and food, are straightforward, as people can frequently encounter flood‐contaminated objects during and after the flood has receded. The dose‐response relationship between IID and the depth of floodwater, direct exposure to floodwater, and levels of water insecurity also supports such direct pathways (Kraay et al., 2020; Reacher et al., 2004; Rosinger, 2018; Schmid et al., 2005). The failure of water treatment plants or contamination from damaged pipes creates indirect pathways, and dose‐response relationships have been estimated by linking the level of consumption from public water supplies after heavy rainfall to the incidence of IID (Atherton et al., 1995; Gong et al., 2019; Levy et al., 2016). Extended power outages after flooding can lead to food spoilage, and consuming spoiled food is another indirect pathway linking floods to an increased risk of IID (Deng et al., 2022; Kosa et al., 2014; Marx et al., 2006).

### 1.2 Remote Sensing Data Sets for Post‐Flood Health Risk Identification

Identifying areas affected by flood‐associated IID is essential for prioritizing interventions, including the provision of sanitation facilities, safe drinking water, food, and medications, to reduce ongoing risks and support recovery efforts. Moreover, near real‐time delineation of flooded areas can support active surveillance for increased IID risk by comparing risk in flooded versus non‐flooded neighborhoods. Such surveillance is crucial for detecting any increases in IID cases and identifying the sources of contamination immediately following flooding and in the subsequent weeks to control disease incidence and spread (Murthy & Christian, 2010; Patz et al., 2000).

Flood‐affected areas can be delineated using direct methods like property damage records and field surveys (Miranda et al., 2021; Oxfam, 2017; USGS, 2018). However, for widespread flooding events, field measurements at every location are impractical (Oxfam, 2017; Stewart & Berg, 2019), involve risks for data collectors, and can be susceptible to errors due to recall and cognitive biases (Guiteras et al., 2015). Indirect methods have proven valuable, such as use of airborne imagery, stream gauge measurements, and hydrological flood models (Anderson et al., 2020; Barcellos & Sabroza, 2001; Klemas, 2015; Shen et al., 2019). However, limitations exist; stream gauges are confined to watercourses, airborne campaigns can be costly and weather‐dependent, and models rely on simulations rather than real‐time data (Klemas, 2015). Satellite observations provide a complementary solution, enabling detection of flooded regions and populations over broad spatial scales (Pickens et al., 2020; Tellman et al., 2021). Satellite platforms that include active radar or passive microwave instruments can capture the ground even under cloud cover (Schumann & Moller, 2015). These satellite‐based methods have their own limitations, such as underestimation in densely urbanized areas and tradeoffs between spatial and temporal resolution (Lillesand & Kiefer, 1994; Schumann & Moller, 2015). Despite these challenges, satellite‐based inundation extents have demonstrated utility in determining health risks during and following flood events (Aggarwal et al., 2025; Dotse‐Gborgbortsi et al., 2022; Ramesh et al., 2022; Sajid & Bevis, 2021; Saulnier et al., 2018).

To protect privacy, health records are often aggregated to administrative boundaries, which necessitates similar aggregation of high‐resolution satellite‐based inundation extents (Fefferman et al., 2005). Two common methods of aggregation used in research on exposure to floods are calculating the flooded area proportion within boundaries (Giannelli & Canessa, 2022; Guiteras et al., 2015; Saulnier et al., 2018), or overlaying inundation extents with population density grids to estimate the number or percentage of affected individuals (Dotse‐Gborgbortsi et al., 2022; Lee et al., 2021; Rolfe et al., 2020; Sajid & Bevis, 2021). The rationality for using these methods, however, is often unclear.

### 1.3 Hurricane Harvey 2017

Hurricane Harvey was a Category 4 hurricane that made landfall in Texas, USA on 26 August 2017, bringing unprecedented levels of rainfall exceeding 60 inches across southeastern Texas (Blake & Zelinsky, 2018). Approximately 300,000 buildings were inundated, and over 1,057 square miles of land were submerged in southeastern Texas (Brakenridge & Kettner, 2017). The hurricane caused 70 fatalities, left 336,000 customers without electricity, and even 3 weeks later, at least 3,900 homes were still without power (Amadeo, 2019; Godfroy & Jonkman, 2017). In addition, 19 water systems and 31 wastewater systems remained offline (Amadeo, 2019; Blake & Zelinsky, 2018). Over 1,500 sanitary sewage overflows resulted in the discharge of more than 150 million gallons of wastewater (Phillips, 2018). Based on the United States Geological Survey (USGS) flood gauges, the flooding in the area persisted until September 13 (USGS, 2020). Reports of IID cases emerged both during and after the flooding, with evacuees in shelters reporting instances, alongside a notable increase in IID‐related emergency department (ED) visits among evacuees who sought refuge in Dallas (Schnall et al., 2020; Stephens et al., 2020).

### 1.4 Objectives

The current work uses two satellite‐based inundation products to demarcate the flooding caused by Hurricane Harvey in 2017 and investigates the association between census tract (CT) flooding and IID‐related ED visits. The utilized inundation data sets are sourced from the Dartmouth Flood Observatory (DFO) and Atmospheric and Environmental Research (AER) (Brakenridge & Kettner, 2017; Galantowicz et al., 2017). The DFO data set is widely used for large flood studies due to its robust integration of satellite and complementary data sources (Brakenridge & Kettner, 2017; Khemani et al., 2025; Schumann et al., 2018; Shen et al., 2019). We selected the DFO and AER products because previous work has demonstrated an association between DFO inundation and flood‐related ED visits, while AER offers superior spatial and temporal resolution (Ramesh et al., 2023). Although the Global Flood Database, a widely applied product based on the DFO flood events, is comprehensive, it does not provide inundation extent for Hurricane Harvey (Tellman et al., 2021). Other data sets, such as the Global Flood Monitoring System (GFMS) and Multi‐Sourced Flood Inventories (MFI), have lower resolution, while Federal Emergency Management Agency (FEMA) flood maps, though widely used, are neither event‐specific nor produced in near real time (FEMA, 2014; Huang et al., 2021; Wu et al., 2014).

Our aim is to evaluate how variations in exposure assessment methods that utilize remotely sensed inundation data can affect the observed association between flooding and IID‐related ED visits. We hypothesize that (a) the estimate of association between flooding and IID‐related ED visits differs based on the underlying inundation extent data sets used for demarcating flooding; and (b) the estimate of association between flooding and IID‐related ED visits differs based on the exposure representation methods (% of land flooded, % of population flooded) used to aggregate flooded areas within census tracts. The results of the study can demonstrate how inundation data sets with varying spatial and temporal resolution can be used for prioritizing locations for interventions following flooding to mitigate water‐borne health risks.

## 2 Materials and Methods

### 2.1 Inundation Data Sets

#### 2.1.1 The Dartmouth Flood Observatory

The Dartmouth Flood Observatory (DFO) at the University of Colorado creates near real‐time inundation extents of flooding events around the world using satellite imagery. Utilizing before‐and‐after comparisons of satellite‐observed surface water in affected regions, the DFO mapped inundation caused by Harvey flooding with a spatial resolution of 190 m (Brakenridge & Kettner, 2017). The primary data source for this inundation mapping effort was a collage of satellite images taken from various parts of Texas on 28 and 29 August, captured primarily by ESA's Sentinel‐1, which provides microwave synthetic aperture radar (SAR) image products that are distributed at a native spatial resolution as high as 5 m × 2 m. Additionally, the data set incorporates Moderate Resolution Imaging Spectroradiometer (MODIS) daily flood data (capable of 250 m resolution) to extend coverage to other flooded days. However, MODIS data are sensitive to cloud cover while Sentinel‐1 radar data are unaffected (Alsdorf et al., 2007; Lillesand & Kiefer, 1994). As a result, the DFO inundation data set offers a spatially complete depiction of flooding only for the days covered by Sentinel‐1 data.

#### 2.1.2 AER FloodScan

Atmospheric and Environmental Research's (AER) FloodScan offers daily inundation extents of widespread inland flooding spanning 1998 to the present. FloodScan's primary inputs are observations from passive microwave remote sensing satellite sensors, including the Advanced Microwave Scanning Radiometer (AMSR2) and Global Precipitation Measurement Microwave Imager (GMI). The satellite sensors capture data at a 22‐km resolution (raw spatial resolution), which is then downscaled to 90 m with an algorithm that considers additional data such as topography, hydrology, and Global Surface Water Explorer data (Galantowicz et al., 2017, 2018). For the current study, Maximum Daily Flood Extent Depiction (MFED) daily inundation extents published by AER specific to the Hurricane Harvey event were acquired, covering the period from 27 August to 9 September 2017 (AER, 2023b). The 90 m product is only produced for specific high‐impact events. However, FloodScan also includes a temporally continuous daily inundation product that is produced at ∼10 km resolution. This product is available from the year 2000 onward and allowed the consideration of historic fractional flooding at the resolution of the coarse product. The FloodScan data set is a proprietary product that must be purchased, whereas the DFO data set is freely available.

**Figure 1:**
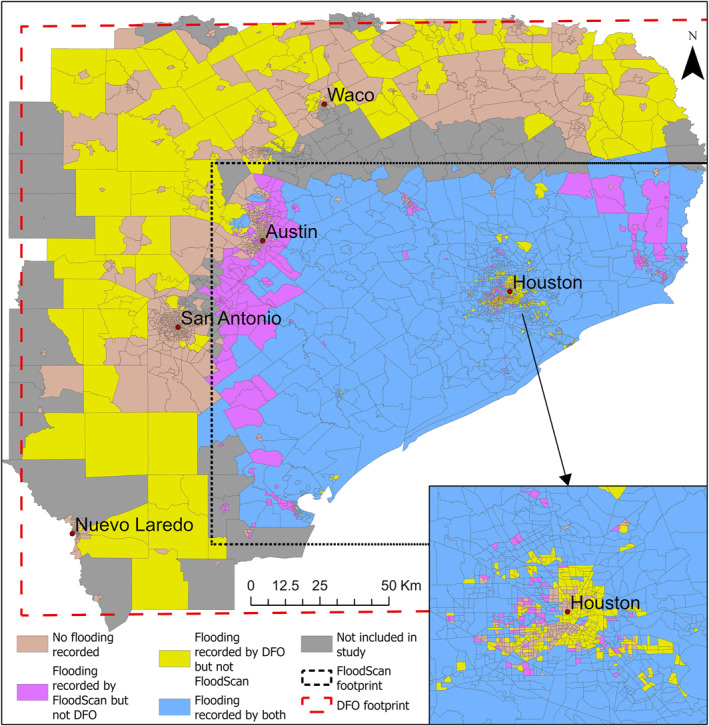
Census tracts in Texas classified as flooded or non‐flooded after Hurricane Harvey landfall based on comparison between the two inundation data sets (DFO and AER FloodScan). Gray shaded census tracts were excluded from the analysis. Due to the small scale of the main map, the symbology for smaller census tracts may not be clearly visible. This is evident in the enlarged map focused on Houston.

Due to the smaller coverage area of the 90 m FloodScan data set compared to that of the DFO data set (Figure 1), census tracts (CTs) were chosen for inclusion in the study if both data sets covered them or if they were within DFO's extended coverage area and were identified as non‐flooded by the low resolution (10 km * 10 km) FloodScan fractional flooding product, which was accessible for the entire state. However, we excluded CTs that were outside the footprint of the high resolution FloodScan product that were categorized as “likely flooded” in the coarse resolution product (the belt of grayed out CTs in Figure 1). The low resolution made it infeasible to calculate exposure metrics used in this study, such as the percentage of land within CT flooded given that the median area of CTs in the study region is 5.2 km^2^. The rationale for incorporating CTs not covered by both data sets is to optimize the utilization of CTs where the flooding status is estimated at the highest available spatial resolution.

### 2.2 Synthetic Population

The synthetic population data set sourced from Adiga et al. (2015) comprises synthetic individuals and households, geographically located using land‐use data and transportation networks, ensuring statistical similarity to the original census. The detailed methodology for constructing and evaluating this data set is discussed elsewhere (Adiga et al., 2015). In brief, the data set was constructed by creating synthetic households using American Community Survey (ACS) data at the block group level and Public Use Microdata Sample (PUMS) information, employing Iterative Proportional Fitting (IPF). These synthetic households were validated by comparing distributions of variables, excluding the ACS variables, in the IPF step. Further locations for these synthetic households were assigned using census cartographic boundary shapefiles defining the number of buildings per block group and satellite imagery‐based data from Microsoft and HERE maps (HERE, 2024) for placement of households based on locations of residential buildings.

### 2.3 Emergency Department Visits

ED visits (both outpatient and inpatient) from July to December of 2016, April to December of 2017, and July to December of 2018 were acquired from the Texas Department of Health and Human Services (DHHS, 2020). The variables included in the ED visit data encompassed patient demographics such as sex, age, race, ethnicity, date of visit, and date of discharge, and census tract geocoded from the patient's street address. Additionally, data on primary, secondary, and other diagnostic codes were available in the International Classification of Diseases (ICD)‐10 format. Individual level ED visits of patients from CTs in the study area totaled 8,482,943 records from 2,697 CTs. From this, 82,972 ED visits from the week before the flood were removed and 414,483 from the month after were analyzed separately (justification provided in study design below), leaving 7,985,488 ED visits. Further excluding 0.03% records with missing values for race/ethnicity or missing or unrealistic values for age (values removed: 132, 167, and 168), 7,983,258 ED visits were available for the final analysis. Non‐Hispanic White patients accounted for 36% of the total ED visits, followed by Hispanic patients at 36%, and Non‐Hispanic Black patients at 20%. Females represented 57% of the visits, while males accounted for 42%. The average age of patients in the data set was 38 years (SD = 24). IID‐related ED visits were distinguished from other ED visits by using ICD codes that were within the A00 to A09 range which collectively covers a variety of bacterial, viral, and protozoal infections that primarily affect the gastrointestinal system. Among the overall ED visits, 58,645 were classified as IID‐related. Approval for this study was granted by the Institutional Review Board (IRB Protocol #18–914) at Virginia Polytechnic Institute and State University and the Institutional Review Board (IRB Protocol #19–024) at the Texas Department of State Health Services.

### 2.4 Exposure Quantification to Census Tracts

The analysis is conducted at the CT level, using CT boundaries from the Census TIGER data (USC, 2010). The utilization of two inundation data sets and two exposure representation methods for aggregating flooded pixels to CTs (zonal statistics) resulted in the formation of four continuous flood exposure variables: the percentage of land within CT flooded according to DFO (*% land flooded*_*DFO*_), percentage of population within CT flooded according to DFO), and likewise for FloodScan (*% land flooded*_*FloodScan*_, *% pop flooded*_*FloodScan*_). Quartiles of exposure representation were also evaluated to examine evidence of non‐linear associations. The percentage of land within each CT flooded was calculated by dividing the inundated area, derived from the inundation data set, by the total non‐water‐covered area based on the National Land Cover Database (Dewitz, 2019). The percentage of population in flooded areas was computed by overlaying the inundation extents on the synthetic population data set that provided household locations (latitude and longitude) and the number of individuals within each household. The spatial variation between the exposure representation derived using the DFO inundation extents is shown as a bivariate map of Figure S1 in Supporting Information S1. The exposure representations showed differences primarily in larger census tracts located far from the coast. In these areas, the percentage of land flooded was high, while the percentage of population affected by flooding was relatively low. In Houston, which was severely impacted by the floods, the deviation between the exposure representations was smaller and appeared more random.

### 2.5 Study Design

A controlled before‐after design (CBA) was implemented to estimate the association between flooding and IID‐related ED visits (Lopez, 2018). This study design allowed estimation of the changes in ED visits in flooded regions compared to the non‐flooded regions during the flood period after accounting for their differences over a baseline period (before and after flood period). The period from 26 August to 13 September 2017, was designated as the flood period, determined by the Hurricane's landfall date and USGS flood gauge data (USGS, 2020). The baseline period covers time before and after the flood period. However, ED visits 1 week before the flood period (19–25 August, 2017) were not included as part of baseline nor the flood period, as evacuation notices could affect the normal ED visit pattern during this period. The 1‐month period after the flood period (ending 13 October) was excluded from the baseline period and analyzed separately to account for potential residual or lag effects immediately following the receding of floodwaters. Of the total overall ED visits, 7,757,619 occurred during the baseline period and 225,639 during the flood period. Among the 58,645 IID‐related ED visits, 1,492 occurred during the flood period.

### 2.6 Statistical Analysis

Modified Poisson regression that incorporates sandwich error estimation was implemented using a generalized estimating equations (GEE) model to estimate risk ratios (RR) for association between census tract flooding and IID‐related ED visits. The GEE model with a log link and Poisson distribution enables direct estimation of RR with robust standard errors for binary outcome (IID‐related vs. non‐IID‐related ED visits), while also allowing to account for clustering at CT level (Yelland et al., 2011; Zou, 2004). Because the outcome is binary and overall ED visits serve as the denominator for risk calculation, an offset term is not required. The model was adjusted for the patient's sex, age, and race/ethnicity. The model's equation is as follows:

(1) $$\log_{e}\left[\pi_{i}\right]=\beta_{0}+\beta_{1}*{\text{flooded}}_{i}+\beta_{2}\;*\;\text{flood}\_\text{period}+\beta_{3}*{\text{flooded}}_{i}*\text{flood}\_\text{period}+\beta_{4}*{\text{sex}}_{i}+\beta_{5}*{\text{age}}_{i}+\beta_{6}*{\text{race}\_\text{ethnicity}\;}_{i}+\beta_{7}*{\text{OP}}_{i}+\text{seasonality}$$

Where π_i_ is the probability of ED visit *i* being an IID‐related ED visit, flooded_i_ denotes the flood exposure of patient's residential CT, “flood_period” is a binary variable to differentiate flood period (1) from the baseline (0). Other variables used for controlling potential confounding are: patient's age (age_i_), sex of the patient (sex_i_, 1 for male and 0 for female), type of visit (OP, 1 for outpatient and 0 for inpatient), and self‐reported race and ethnicity of patient (race_ethnicity_i_) which was categorized as non‐Hispanic White, non‐Hispanic Black, Hispanic, and Other (American Indian, Asian or Pacific Islander, Aleut, and Eskimo). Seasonality was controlled by adding year, month, and day of week as indicator variables (Bhaskaran et al., 2013). We also conducted a supplementary analysis incorporating the Centers for Disease Control and Prevention/Agency for Toxic Substances and Disease Registry Social Vulnerability Index (CDC/ATSDR SVI) at the census tract level, categorized into quartiles (CDC, 2021c). The exponentiation of the coefficient of the interaction term (exp [*β*_3_]) gives the risk ratio (RR) estimate for association between flooding and IID‐related ED visits, by comparing risk of IID‐related ED visits in flooded regions to that of non‐flooded regions during the flood period, while accounting for the baseline risk difference between those regions. In Equation 1, the exp (*β*0 + *β*1 + *β*2 + *β*3), represent the risk of IID‐related ED visits in flooded regions during the flood period, exp (*β*0 + *β*2) corresponds to risk in non‐flooded regions during the flood period, and exp (*β*1) corresponds to the ratio of baseline risk between those regions.

Each of the four exposure variables discussed above were fit separately in the model specified in Equation 1. The Quasi‐Likelihood under the Independence Model Criterion (QIC) values of these fitted models were compared to check which flood variable provided a better fit (Pan, 2001). Furthermore, bias‐corrected and accelerated (BCa) confidence intervals (CI) were computed using 1000 samples with replacement to determine if there were significant differences in the RR between the four different flood exposure variables (DiCiccio & Efron, 1996; Kropko & Harden, 2020). RR estimates were regarded significantly different if the bootstrapped 95% confidence interval for the difference between them on the natural logarithm scale (ln{RR}) excluded zero.

When the flood exposure variable was taken as a binary variable in the above equation, non‐zero values for the CT were considered flooded for both the percentage of land and the percentage of population flooded within the CT. A sensitivity analysis was conducted by setting the threshold for binary classification of “flooded” based on the historical average percentage of land flooded (for every 10 km^2^) during summer (June to September) in the study region between 2000 and 2016 (AER, 2023a). In a separate analysis, the neighbors of the flooded CTs were classified as a distinct category to assess whether the risk of IID‐related ED visits was elevated in the bordering CTs as well. This was done for two reasons: (a) to address potential inaccuracies resulting from the downscaling process of FloodScan data set, which could overlook flooding in specific CTs when interpolating near border areas, and (b) to investigate the potential indirect and wider scale effects of flooding in one CT on its neighboring ones (e.g., shared drinking water, sanitation and food sources).

## 3 Results

### 3.1 Differences Between DFO and FloodScan Inundation Data Sets

Among the 2,697 CTs in the study area, 860 CTs experienced flooding according to both DFO and FloodScan inundation extents. Likewise, 1,103 tracts were identified as non‐flooded by both sources, while discrepancies were present in 734 CTs (27%) (Figure 1). The agreement between DFO and FloodScan inundation extents as assessed by Cohen's Kappa (Cohen, 1960) was 0.45. When considering if any population/households in the CT flooded, agreement between the inundation extents was slightly higher (*κ* = 0.46). When comparing the agreement between the two representations of exposure within a CT (any land flooded vs. any of the population flooded), a high level of agreement was found: *κ* = 0.84 for DFO and *κ* = 0.79 for FloodScan. Based on DFO (FloodScan) inundation extents, 210 (278) CTs experienced flooding in part of their land without any population or households within the flooded areas.

The Pearson correlation between the inundation data sets was moderate (*ρ* = 0.5) when comparing using % land flooded and lower (*ρ* = 0.3) when using % population flooded (Table 1). However, the correlation between the exposure representations (i.e., correlation between % land flooded and % population flooded) was strong within the data sets: 0.86 for FloodScan and 0.8 for DFO. A supplementary correlation analysis that weighted by census tract population produced similar results (Tables S1 and S2 in Supporting Information S1).

**Table 1:** Correlation Between Inundation Data Sets (FloodScan and DFO) Using Different Flood Exposure Representations (% Land Within Census Tract Flooded and % Population Within the Census Tract Flooded)

	% Land flooded_DFO_	% Land flooded_FloodScan_	% Pop flooded_DFO_	% Pop flooded_FloodScan_
% land flooded_DFO_	1	–	–	–
% land flooded_FloodScan_	0.49	1	–	–
% pop flooded_DFO_	0.80	0.35	1	–
% pop flooded_FloodScan_	0.33	0.86	0.30	1

### 3.2 Differences Between Risk Ratio Estimates

#### 3.2.1 Comparison Across Inundation Extent Data Sets

**Figure 2:**
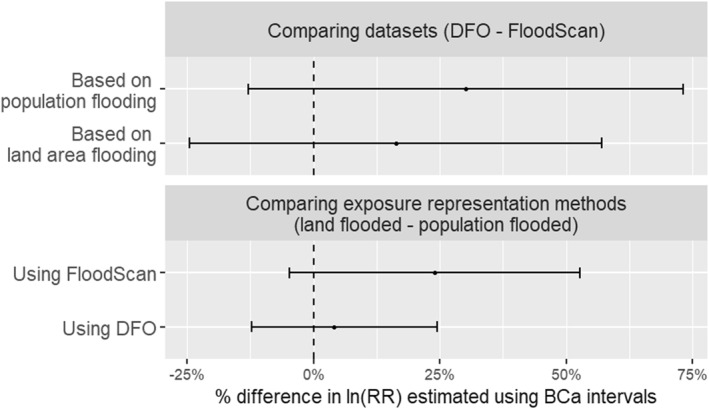
The percentage difference between the ln(RR) estimated using the two different inundation data sets and the two exposure representations, calculated from 95% BCa confidence intervals.

Using any land flooded as the exposure representation (binary exposure), as per DFO inundation extents, the risk of IID‐related ED visits in the flooded CTs was 1.28 times (95% CI: 1.15, 1.43) the risk in the non‐flooded CTs, after adjusting for differences over the baseline period (Table 2). Using the FloodScan inundation extents, in place of DFO, the risk ratio for the association was 1.23 (95% CI: 1.10, 1.34). The difference between the two risk ratios was not statistically significant, as the 95% BCa CI ranges from −0.06 to 0.14 and includes 0 (Figure 2). Even when the exposure representation was altered to population within the CT flooded, the difference of ln(RR) estimated between the two inundation data sets was not statistically significant (BCa CI: −0.03, 0.17) (Figure 2).

**Table 2:** Risk Ratios for Association Between IID‐Related ED Visits and Flooding Delineated Using DFO and FloodScan Data Sets Using Different Binary or Continuous Flood Exposure Representation Methods (Any Land Within CT Flooded and Any Population Within the CT Flooded)

		Binary		Continuous^a^	
		RR (95% CI)	QIC	RR (95% CI)	QIC
Land flooded	DFO	1.28 (1.15, 1.43)	545,009	1.06 (1.01, 1.10)	545,136
	FloodScan	1.23 (1.10, 1.37)	544,890	1.05 (1.02, 1.09)	545,167
Population flooded	DFO	1.26 (1.13, 1.41)	545,040	1.06 (0.98, 1.14)	545,171
	FloodScan	1.18 (1.05, 1.32)	545,069	1.03 (0.99, 1.07)	545,172

^a^ Per 10% increase.

#### 3.2.2 Comparison Across Exposure Representation Methods

When comparing the difference in ln(RR) between two exposure representations (land flooded vs. population flooded) using DFO inundation extents, the estimates were not significantly different (Figure 2). This was also true when using the FloodScan data set; however, the estimate was marginally higher when using population flooded (BCa CI: −0.01, 0.11).

Sensitivity analysis of using AER historic inundation extents to create a threshold based on local historic seasonal floodings in the region for binary classification did not change the results (Figure S2 in Supporting Information S1). The supplementary analysis, which examined the risk ratios for the lag period of 1 month following the flood (14 September to 13 October 2017), found none of the estimates corresponding to the binary exposure variables to be statistically significant (Table S3 in Supporting Information S1). Supplementary analysis conducted by including CDC/ATSDR SVI as a census tract level covariate did not change the estimated associations for either data set or exposure representation (Table S4 in Supporting Information S1).

When using the percentage of land flooded in the models (as a continuous variable), a 10% increase was associated with a 6% (1%–10%) increase in IID‐related ED visits. The association was not significant when using the percentage of population flooded from DFO or FloodScan data sets (Table 2); when exposure quartiles were used, however, a significant increase was observed in the second, third, and fourth quartiles of DFO and the second and third quartiles of FloodScan (Figure S3 in Supporting Information S1). Whether flooding was used as binary or continuous variable in the models, assigning flood exposure to CTs based on the land flooded method resulted in better model fit, as measured by QIC (Table 2).

**Figure 3:**
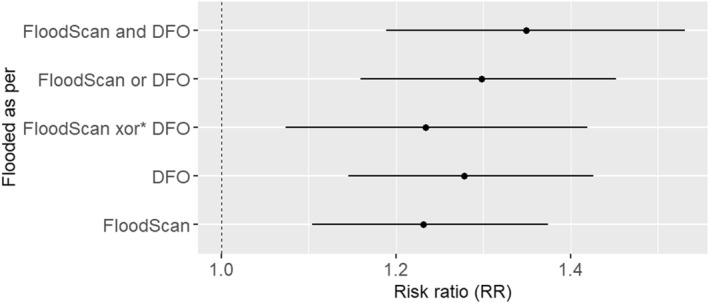
Risk Ratio for association between IID‐related ED visits and flooding mapped using a single inundation data set or a combination of the two data sets. *xor—RR for census tracts that are marked flooded by either data set but not both. “or”—RR for census tracts that are marked flooded by either data set. “and” = RR for census tracts that are marked flooded by both data sets.

Since inundation extents from both data sets indicated elevated risk in their respective identified CTs with any land flooded, we analyzed if the risk was elevated in the regions of disagreement compared to the mutually agreed non‐flooded CTs (Figure 1). Comparing the CTs that were discordant (FloodScan xor DFO) to that of the non‐flooded CTs, it was found that the risk of IID‐related ED visits in these CTs was 1.25 (1.08, 1.45) times the risk among the non‐flooded CTs (Figure 3). Also, the model fitness was the best (QIC = 544,716) when FloodScan and DFO were jointly used to identify the flooding of land within the CTs (considering flooded if either data set indicates flooding). Under this classification (FloodScan or DFO) the estimated RR was 1.30 (1.16, 1.45) (Figure 3). When CTs were classified as flooded only if both data sets agreed on flooding (FloodScan and DFO), the estimated RR was 1.35 (95% CI: 1.19, 1.53) (Figure 3).

### 3.3 IID Risk Among Neighbors of Flooded Census Tracts

Evaluating the risk of IID‐related ED visits in non‐flooded neighbors of the flooded CTs according to the DFO inundation extents, the risk for IID‐related ED visits was not significantly elevated compared to the non‐flooded CTs (RR = 1.08; 95% CI: 0.92, 1.27). However, the risk significantly elevated among the neighbors of FloodScan‐identified flooded CTs (RR: 1.19; 95% CI: 1.01, 1.41) compared to the non‐flooded CTs.

## 4 Discussion

This study compares two remotely sensed inundation data sets (DFO and FloodScan) with varying spatial and temporal resolutions, to determine if the association between CT flooding and IID‐related ED visits differs depending on the inundation data set used, while also evaluating the difference between flood exposure representation methods in terms of land flooded versus population flooded. In short, the FloodScan data set is generated from satellite sensors with high temporal resolution but low spatial resolution (22 km), then enhanced using ancillary data on landscape characteristics to achieve high spatial resolution (90 m). On the other hand, the DFO data set utilizes data from satellite sensors with high spatial resolution (∼10–250 m) but low temporal resolution, which is then adjusted to reduce classification errors to get the final inundation extents at a slightly lower spatial resolution (190 m). For the period during and after Hurricane Harvey, the 90 m FloodScan product offers both higher temporal and higher spatial resolution than DFO, but its flooded area estimates are derived from the downscaling model rather than being directly observed in high resolution satellite imagery. Despite these differences between the inundation data sets, both the data sets were able to capture the elevated risk of IID‐related ED visits associated with flooding and we did not observe statistically significant differences in the estimates between the inundation data sets. This could be because, although the data sets disagreed in 27% of the CTs, the classification disagreement between flooded and non‐flooded was lower in heavily flooded CTs than in those with lower levels of flooding (Table S5 in Supporting Information S1). The high level of agreement observed between the two exposure representations (*κ* ≥ 0.79) is expected, since census tracts cannot have a flooded population without also experiencing flooded land. This may explain the similarity in association estimates across the two exposure representations.

Using FloodScan, we found that the risk of IID‐related ED visits was also elevated among adjacent CTs of flooded CTs. This was not observed when using the DFO data set. This could be due to under classification caused by downscaling from 22 km satellite observation to 90 m grids, which might overlook flooding in specific CTs when interpolating near borders, as the median size of the CTs is 5.64 km^2^. In fact, 44% of the non‐flooded neighbors of the flooded FloodScan regions were labeled as flooded by DFO data set. Therefore, when employing low raw spatial resolution products such as FloodScan to identify CTs for disaster response, neighbors of flooded CTs should be evaluated separately or combined with the exposed group, as risk for IID may also be heightened in these areas.

Overall, using both the inundation data sets together to classify CT land areas resulted in the best model for estimating IID‐related ED visit risk. From an application standpoint, we observed that the risk of IID associated with flooding was also elevated in areas where the data sets disagreed, emphasizing the importance of including these areas in recovery planning efforts. Inundation extent mapped with high raw spatial resolution (like DFO) can reduce mixed pixel problems—classification difficulties caused by different land surfaces within the same grid square—thus enabling more accurate detection of smaller flooded areas (Dotse‐Gborgbortsi et al., 2022; Schumann et al., 2018). However, this finer resolution often comes with lower temporal resolution and a reduced spatial extent of observation, meaning they might not capture peak flood extents in all affected areas and could underestimate exposure. The high temporal resolution of the FloodScan data set offers daily flood estimates but might create uncertainties due to the downscaling component. Integrating high temporal resolution data sets with high raw spatial resolution data sets might be a more effective approach for disaster recovery interventions. This aligns with Schumann et al. (2018), who state that “the full potential of EO data (satellite observations data) is unlocked when many different missions and sensors … are combined in an intelligent way.”

The lack of difference in the estimated association between the inundation data sets suggests that, from a risk assessment perspective, remote sensing data sets with varying spatial and temporal resolutions may not substantially impact the estimate of association. However, areas where the data sets disagreed showed elevated risk of IID‐related emergency department visits. This highlights the importance of combining high‐temporal and high‐spatial resolution products to avoid overlooking regions with elevated health risks associated with flooding for disaster management and recovery planning.

Following floods, recovery‐based interventions are essential in the identified affected region to reduce the risk of IID (Jafari et al., 2011; Lemonick, 2011; Murthy & Christian, 2010). This includes distributing chlorine tablets or bottled water to improve access to safe drinking water, with clear communication to address hesitancy toward self‐chlorinated water (Crider et al., 2023; Mitro et al., 2019). Similarly, food safety interventions include managing flood‐exposed food, providing displaced populations with safe food options, and communicating that consumers should only consume fully cooked meals or food kept at safe time and temperature controls (CDC, 2021a). Access to sanitation demands should also be achieved by installing temporary public sanitation facilities and handwashing stations. Additionally, vector control measures, such as eliminating standing water containers and indoor residual spraying, are crucial in preventing disease spread (Jafari et al., 2011; Lemonick, 2011). Lastly, reinstating primary healthcare services in the affected regions and ensuring quick fulfillment of medication demands are vital for early diagnosis and treatment (Adalja et al., 2014; Lee et al., 2015).

In the current study, compared to CT land flooded, further refining CT based exposure estimates to consider locations of residences using the synthetic population data set did not improve model fit when used with either product. The exposure representation method that incorporated the residential location within CT estimated the IID risk to be 22% lower (−4%–54%) compared to the land flooded measure, which might suggest that non‐residential flooding around residential areas could also influence the risk of IID. This result is consistent with the hypothesis that primary exposure pathways of infectious agents causing IID following floods are more centralized (Ramesh et al., 2023). These pathways include contamination of drinking water sources, disruptions to drinking water connectivity, power outages, and healthcare access (Levy et al., 2016; Marx et al., 2006).

Incorporating estimates of residential locations may introduce an additional layer of uncertainty to the models, compounding the existing uncertainty in the flood layer, which could increase the overall uncertainty around the risk ratio estimate. A more direct approach is to measure exposure as the percentage of land flooded near home locations, which will preclude the need for population grid products. In the current analysis, the estimated association for a linear increase in flood exposure was not significant when flood exposure was represented as percentage of population flooded using residential location estimates.

These findings highlight that researchers should be careful in selecting the method of flood representation for their studies and should consider the additional uncertainties introduced by population density grids, and the pathways they aim to capture before choosing an exposure measure for estimating health risks during and following floods. Flood is a hazard and estimating its health risk may require defining/measuring exposure in a way that captures population that was affected by the hazard either directly or indirectly or both (Saulnier et al., 2021). For example, respondents whose home was not directly flooded but reported their residential block as flooded still reported illness attributed to the flooding (Miranda et al., 2021; Ramesh et al., 2023). Therefore, overlaying inundation extents on population grids may not be the most accurate method for understanding the health risks associated with flooding as it might not capture the indirect effects.

The RR estimated using DFO is comparable to our previous work with the same data set but is slightly higher, which can be attributed to the exclusion of certain CTs in the current study due to differences in the data extent between the two data sets being compared (Ramesh et al., 2021). Similar to our finding in the current study, Dotse‐Gborgbortsi et al. (2022) analyzed the relationship between two satellite‐derived flood products with diarrhea cases following a dam‐mediated flooding event and reported the higher resolution Landsat better predicted outpatient attendance than MODIS (based on AIC). That study found a fourfold increase in diarrhea‐related outpatient visits for each unit percentage increase in flood area within the facility catchment area based on Landsat data (Dotse‐Gborgbortsi et al., 2022). However, the study did not compare and evaluate if the estimates obtained from the two satellite products were different. Other research has also shown associations between remotely sensed inundation data sets and diarrheal disease using MODIS satellite observations, such as increases up to 3 months after flooding in Pakistan (Sajid & Bevis, 2021); and increases in the incidence of diarrhea in Cambodia flooded areas (estimated flood extents measured in 10 square kilometer units) (Saulnier et al., 2018).

This study has several important limitations. It captures only cases of IID that led to an ED visit. Since gastrointestinal symptoms often do not result in seeking healthcare, the present analysis only reflects severe cases. Additionally, during active flooding, the likelihood of underreporting may increase in flooded regions for any health outcome due to evacuation and limitations in accessing healthcare facilities (Dotse‐Gborgbortsi et al., 2022; Ramesh et al., 2021), and hence the actual association may be stronger than what was captured in the present analysis. The current analysis also does not account for population displacement in the region during the flooding, due to the lack of comprehensive data on large‐scale evacuations and limited information on when people returned after evacuating. We are also limited in our data product comparison by the fact that FloodScan only released 90 m resolution analysis for a core affected area during Hurricane Harvey, and this area was smaller than the area of DFO analysis.

## 5 Conclusion

Both DFO and FloodScan inundation extents indicated an increase in IID‐related emergency department visits during the flood period within the identified flooded CTs following Hurricane Harvey. It is notable that both satellite‐derived products capture this elevated risk, despite their moderate disagreement in the identification of flooded areas, and despite the known challenges of associating a satellite‐observed flood occurrence with personal exposures relevant to IID. To maximize public health benefits, high‐resolution data sets with limited temporal coverage should be combined with lower‐resolution data sets that provide more frequent observations when assessing health risks and prioritizing support after flood events. Our study highlights considerations and cautions in using remotely sensed inundation data sets with varying spatial‐temporal resolutions for targeting communities for recovery measures. This can assist disaster management teams in understanding the importance of inundation data set specifications and where to focus interventions related to healthcare access or safe drinking water advisories based on these specifications.

## Conflict of Interest

The authors declare no conflicts of interest relevant to this study.

## Supporting information

Supporting Information S1

## Data Availability

The CT level exposure estimates computed using the DFO inundation data and NLCD, R code for identifying neighborhood structure among the CTs, python code for data cleaning and statistical analysis are available at Zenodo repository via https://doi.org/10.5281/zenodo.18057565 with Creative Commons Attribution license 4.0 (Ramesh et al., 2025). The FloodScan inundation extent is proprietary and therefore cannot be shared, but it is available for purchase through AER (AER, 2023b). Emergency department visit data are confidential and not publicly accessible; however, researchers may request access by contacting the Texas Department of Health and Human Services (DHHS, 2020).
