# Supplementary material for: Evaluation of Remotely Sensed Inundation Data Sets to Estimate Flood‐Associated Emergency Department Visits After Hurricane Harvey
Source: Geohealth. 2025 Dec 27;10(1):e2025GH001516. doi: 10.1029/2025GH001516 (PMC12743201; doi:10.1029/2025GH001516)
Supplement: Supplementary file 1 — Supporting Information S1 [file GH2-10-e2025GH001516-s001.docx]

*GeoHealth*

Supporting Information for

**Evaluation of Remotely-Sensed Inundation Datasets to Estimate Flood-Associated Emergency Department Visits after Hurricane Harvey.**

Balaji Ramesh^1^, Julia M. Gohlke^2^, Benjamin Zaitchik^3^, Ayaz Hyder^1^, Jeffrey J. Wing^1^, Gia Barboza-Salerno^4^, Samarth Swarup^5^

^1^ College of Public Health, The Ohio State University, Columbus, Ohio, United States

^2^ Population Health Sciences, Virginia Maryland College of Veterinary Medicine, Virginia Tech, Blacksburg, Virginia, United States

^3^ Morton K. Blaustein Department of Earth and Planetary Sciences, Johns Hopkins University, Baltimore, Maryland, United States

^4^ College of Social Work, The Ohio State University, Columbus, Ohio, United States

^5^ Biocomplexity Institute and Initiative, University of Virginia, Charlottesville, Virginia, United States

**Contents of this file**

Tables S1 to S5

Figures S1 to S3

**Introduction**

This document provides supplementary tables and figures presenting the results of sensitivity analyses, along with additional information that supports the discussion in the main article.

Table S1. Population weighted correlation between inundation datasets (FloodScan and DFO) using different flood exposure representations (% land within census tract flooded and % population within the census tract flooded). The weighted correlation were similar to the unweighted correlation presented in table 1.

|  | % land flooded_DFO_ | % land flooded_FloodScan_ | % pop flooded_DFO_ | % pop flooded_FloodScan_ |
| --- | --- | --- | --- | --- |
| % land flooded_DFO_ | 1 | - | - | - |
| % land flooded_FloodScan_ | 0.52 | 1 | - | - |
| % pop flooded_DFO_ | 0.83 | 0.38 | 1 | - |
| % pop flooded_FloodScan_ | 0.36 | 0.85 | 0.31 | 1 |

Table S2. Population Weighted Cohen’s kappa for the agreement between DFO and FloodScan inundation extents.

| **Dataset/exposure representation** | **Comparison** | **kappa** |
| --- | --- | --- |
| Land flooded | DFO vs FloodScan | 0.50 |
| Population flooded | DFO vs FloodScan | 0.51 |
| DFO | Land flooded vs Population flooded | 0.87 |
| AER | Land flooded vs Population flooded | 0.79 |

Table S3. Risk ratio (RR) for association between IID-related ED visits and flooding during the month following the flood period (September 14th to October 13th) delineated using DFO and FloodScan inundation datasets using different binary flood exposure representation methods (any land area within census tract flooded and any population in the census tract flooded). 95% confidence intervals are provided in parentheses. Model fitness measured using Quasi-likelihood under the Independence Model Criterion (QIC).

| Exposure representation | Dataset | RR (95% CI) | QIC |
| --- | --- | --- | --- |
| Land flooded | DFO | 1.00 (0.93, 1.09) | 571004 |
|  | FloodScan | 0.99 (0.91, 1.07) | 570875 |
| Population flooded | DFO | 1.00 (0.92, 1.09) | 571037 |
|  | FloodScan | 0.98 (0.90, 1.07) | 571064 |

Table S4. Risk ratios for association between IID-related ED visits and flooding delineated using DFO and FloodScan datasets using different binary or continuous flood exposure representation methods (any land within CT flooded and any population within the CT flooded) after adjusting for CDC/ATSDR SVI in addition to covariates in model equation 1. Results remained unchanged from the main analysis not adjusting for SVI (Table 2).

|  | **Flooded as per** | **RR (95% CI)** |
| --- | --- | --- |
| Land flooded | DFO | 1.28 (1.14, 1.42) |
|  | FloodScan | 1.23 (1.10, 1.37) |
|  | FloodScan or DFO | 1.30 (1.16, 1.45) |
|  | FloodScan and DFO | 1.35 (1.19, 1.53) |
| Population flooded | DFO | 1.26 (1.13, 1.41) |
|  | FloodScan | 1.17 (1.05, 1.32) |

Table S5. Cross-tabulation showing the percentage of census tracts (CTs) within each cross-category of tertile flood classification (low, moderate, high) based on DFO and FloodScan data. The quantile intervals are indicated in parentheses.

| N=2,697 CTs | FloodScan (% land flooded) | | | |
| --- | --- | --- | --- | --- |
| DFO (% land flooded) | Non flooded | Low (0,1.8] | Moderate (1.8,10.7] | High (10.7,99.3] |
| Non flooded | 40.9% | 6.8% | 3.1% | 2.5% |
| Low (0,3.0] | 8.1% | 3.2% | 2.7% | 1.6% |
| Moderate (3.0,12.6] | 5.3% | 3.1% | 4.6% | 2.6% |
| High(12.6,92.5] | 1.4% | 1.7% | 4.4% | 8.1% |


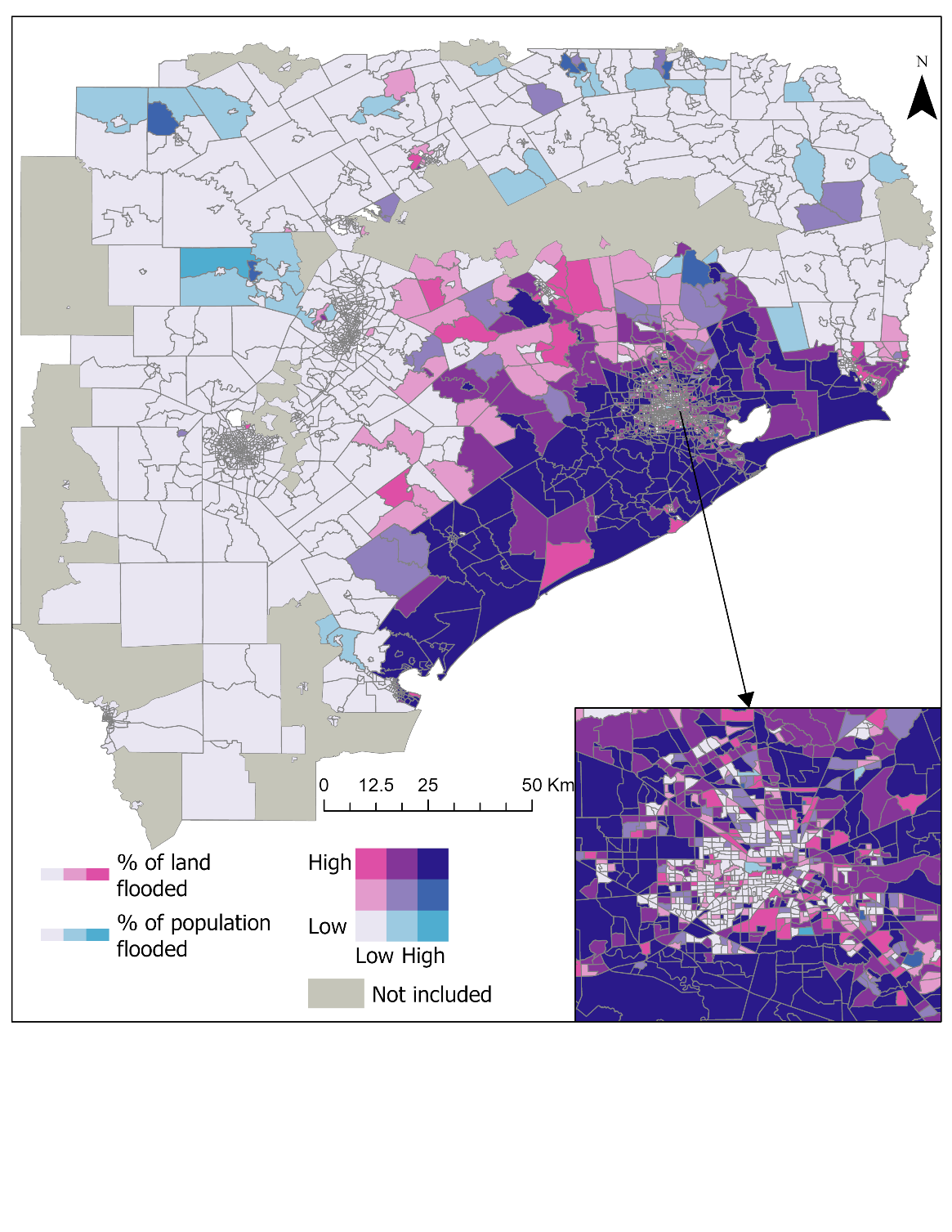


Figure S1. Bivariate map showing the variation between % of land flooded and % of synthetic population flooded within each census tract in the study area. Categories: Low <1%, Moderate >=1% to <5%, High >=5%.


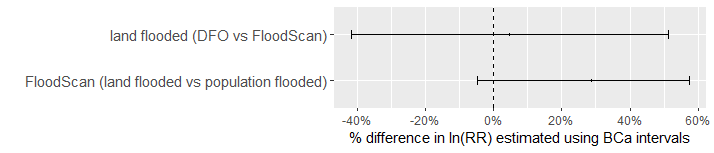


Figure S2. Percentage difference in ln(RR) estimates: between DFO and FloodScan for land flooded (top), and (2) between land flooded and population flooded for FloodScan. For FloodScan, the binary exposure, land flooded was calculated by comparing the percentage of land flooded during the flood period to the historical average during June–September from 2000 to 2016. 95% Bias-Corrected and Accelerated (BCa) bootstrap confidence intervals are shown. The results remained consistent with those discussed in Figure 2.


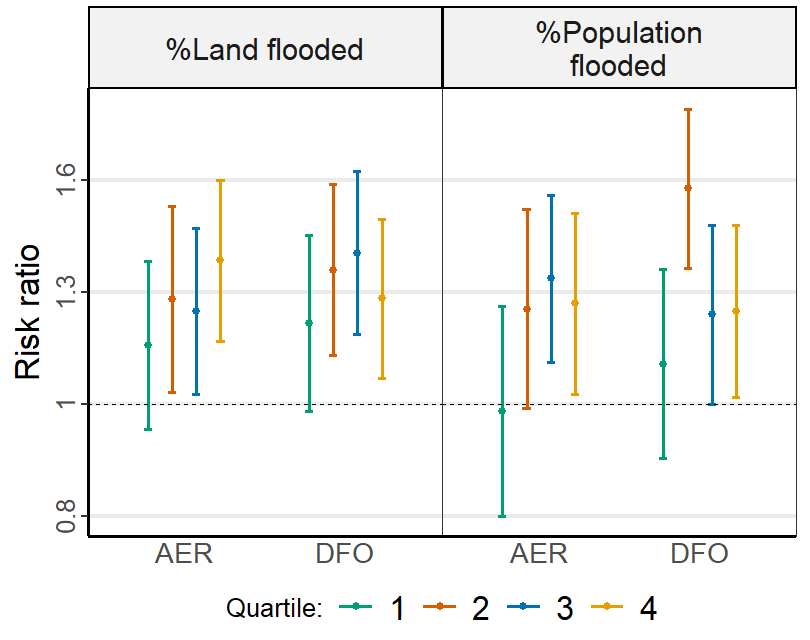


Figure S3. Risk ratio for association between IID-related ED visits and flood exposure expressed as % of land flooded or % of population within CT flooded categorized into quartiles. Reference category was all non-flooded CTs where the percentage was 0.
